# Reevaluation of bromodomain ligands targeting BAZ2A

**DOI:** 10.1002/pro.4752

**Published:** 2023-09-01

**Authors:** Giulia Cazzanelli, Andrea Dalle Vedove, Eleonora Parolin, Vito Giuseppe D'Agostino, Andrea Unzue, Cristina Nevado, Amedeo Caflisch, Graziano Lolli

**Affiliations:** ^1^ Department of Cellular, Computational and Integrative Biology—CIBIO University of Trento Trento Italy; ^2^ Department of Chemistry University of Zürich Zürich Switzerland; ^3^ Department of Biochemistry University of Zürich Zürich Switzerland

**Keywords:** BAZ2 bromodomain, prostate cancer, protein crystallography, small molecule inhibitors

## Abstract

BAZ2A promotes migration and invasion in prostate cancer. Two chemical probes, the specific BAZ2‐ICR, and the BAZ2/BRD9 cross‐reactive GSK2801, interfere with the recognition of acetylated lysines in histones by the bromodomains of BAZ2A and of its BAZ2B paralog. The two chemical probes were tested in prostate cancer cell lines with opposite androgen susceptibility. BAZ2‐ICR and GSK2801 showed different cellular efficacies in accordance with their unequal selectivity profiles. Concurrent inhibition of BAZ2 and BRD9 did not reproduce the effects observed with GSK2801, indicating possible off‐targets for this chemical probe. On the other hand, the single BAZ2 inhibition by BAZ2‐ICR did not phenocopy genetic ablation, demonstrating that bromodomain interference is not sufficient to strongly affect BAZ2A functionality and suggesting a PROTAC‐based chemical ablation as an alternative optimization strategy and a possible therapeutic approach. In this context, we also present the crystallographic structures of BAZ2A in complex with the above chemical probes. Binding poses of TP‐238 and GSK4027, chemical probes for the bromodomain subfamily I, and two ligands of the CBP/EP300 bromodomains identify additional headgroups for the development of BAZ2A ligands.

## 1 INTRODUCTION

Bromodomain adjacent to zinc finger domain proteins 2A and 2B (BAZ2A and BAZ2B) are components of the different ISWI (Initiation SWItch) chromatin remodeling complexes nucleolar remodeling complex (NoRC) and BAZ2B‐containing remodeling factor (BRF), respectively, (Oppikofer et al., 2017). The NoRC regulates rDNA accessibility (Strohner et al., 2001; Zhou & Grummt, 2005). Instead, chromatin regions targeted by the BRF are still largely undefined, although BAZ2B has recently been reported to enhance stemness and multipotency in hematopoietic cells (Arumugam et al., 2020) and to repress the expression of mitochondrial genes (Yuan et al., 2020). Two BAZ2 chemical probes have been developed, namely GSK2801 and BAZ2‐ICR, which could be used to better define the physiological role of BAZ2A and BAZ2B and to evaluate their involvement in pathological contexts (Chen et al., 2016; Drouin et al., 2015). These two chemical probes have been used in a study assessing the potential synergism of kinase inhibitors and epigenetic regulators with BET (bromodomain and extra‐terminal domain) inhibitors in triple‐negative breast cancer (TNBC; Bevill et al., 2019). The authors showed that GSK2801 and BAZ2‐ICR did not affect TNBC cells but that concomitant inhibition of BET, BAZ2, and BRD9 bromodomains synergistically elicited complete growth suppression. This effect could be achieved by combined treatment with either JQ1 (BET inhibitor) and GSK2801 (BAZ2 and BRD9 inhibitor) or with JQ1, BAZ2‐ICR (BAZ2‐specific) and BI‐9564 (BRD9 inhibitor), highlighting the possible therapeutic relevance of “selectively promiscuous” bromodomain inhibitors.

BAZ2A was found overexpressed in prostate cancer (PCa) cells and correlated with the tumor stage. It contributes to migration, invasion and stemness of metastatic and recurrent PCa (Gu et al., 2015). We characterize the effects of GSK2801 and BAZ2‐ICR on two PCa cell lines, either androgen receptor (AR) positive or negative. GSK2801 reduces cellular growth in both cell lines and in all conditions tested, while BAZ2‐ICR is effective in AR‐positive 22Rv1 3D spheroids, but not in PC3 in the same setting. Co‐treatment of PCa cells with BAZ2‐ICR and BI‐9564 does not reproduce the effects observed with GSK2801 suggesting off‐target confounding factors for this compound. We also show that BAZ2‐ICR is not able to recapitulate the effects observed with BAZ2A genetic ablation; this was also recently demonstrated by a different research group reporting that BAZ2‐ICR does not affect the proliferation of heterogeneous PCa cell population while being effective on PCa cancer stem cells (Peña‐Hernández et al., 2021). We finally report the crystallographic structures of GSK2801 and BAZ2‐ICR in complex with the BAZ2A bromodomain; those structures could guide the evolution of these inhibitors in PROteolysis TArgeting Chimera (PROTACs) or other degraders exploiting different technologies (Békés et al., 2022; Ding et al., 2020; Paiva & Crews, 2019) to chemically reproduce the observed efficacy of BAZ2A genetic ablation. Notably, a GSK2801 PROTAC, through careful optimization of the linker region, could also relieve the reported off‐target effects, gaining specificity through the cooperative contribution of surface interactions between the bromodomain and the E3 ligase (Bondeson et al., 2018; Gadd et al., 2017; Smith et al., 2019). At the same time, we investigated the binding mode to BAZ2A of chemical probes developed against the CECR2‐GCN5‐PCAF‐BPTF bromodomain subfamily I (Cat Eye syndrome chromosome region, candidate 2—general control non‐derepressible 5—p300/CREB binding protein associated factor—bromodomain and Plant HomeoDomain finger‐containing transcription factor) and of inhibitors of the p300/CBP bromodomains. The newly determined crystallographic structures, and their comparison with those already deposited of the same inhibitors in complex with their target bromodomains, offer useful pharmacophoric insights for the future exploitation of these scaffolds toward BAZ2 proteins.

## 2 RESULTS AND DISCUSSION

The Structural Genomics Consortium developed several chemical probes for a variety of bromodomains (https://www.thesgc.org/chemical-probes/epigenetics; Wu et al., 2019). BAZ2‐ICR is the most potent BAZ2A inhibitor developed so far (*K*_D_ = 109 nM), also inhibiting BAZ2B with similar potency (*K*_D_ = 170 nM; Drouin et al., 2015); weaker activity is observed for the inhibition of the CECR2 bromodomain (*K*_D_ = 1.55 μM). The only other BAZ2 chemical probe available is GSK2801, with *K*_D_ = 257 and 136 nM for BAZ2A and BAZ2B, respectively, (Chen et al., 2016); it also inhibits BRD9 (*K*_D_ = 1.2 μM) and TAF1L (*K*_D_ = 3.2 μM) bromodomains.

**FIGURE 1:**
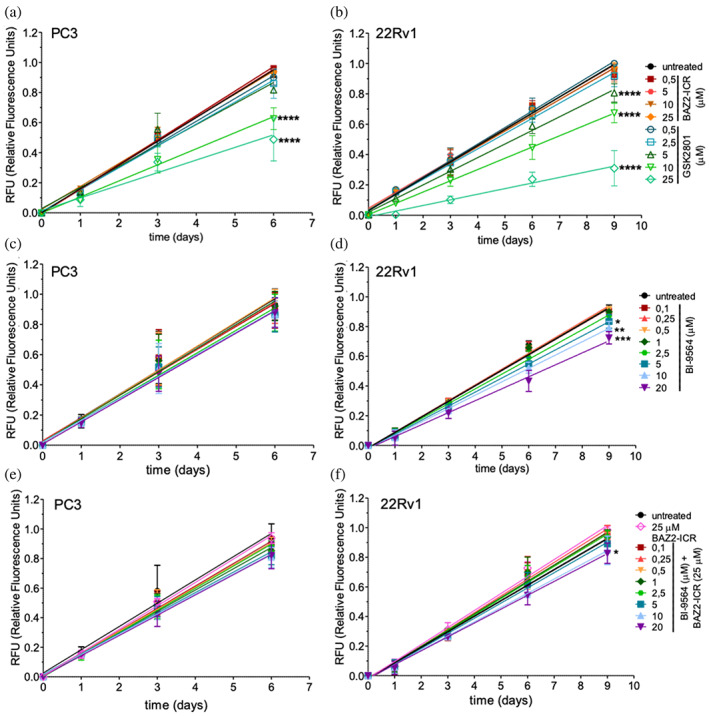
Effect of GSK2801 and BAZ2‐ICR on PCa cells. Each point represents the mean ± SD of 3–5 independent replicates, depending on the cell lines and the treatment. For each replicate, three wells/conditions were seeded. Lines are obtained using the linear regression analysis of Graphpad Prism software. The treatment was considered to have an effect when slopes for treated conditions were statistically different from the untreated. ns = *p* > 0.05, *p < 0.05, **p < 0.01, ***p < 0.001, ****p < 0.0001. (a), (b) GSK2801, but not BAZ2‐ICR, reduces 2D growth of PC3 (AR‐negative) and 22Rv1 (AR‐positive) cells. (c), (d) The BRD9 inhibitor BI9564 shows limited activity only on 22Rv1 cells. (e), (f) Cotreatment with BAZ2‐ICR and BI9564 does not reproduce the effects observed with GSK2801.

BAZ2A depletion by siRNA was reported to slow proliferation rates and impair migration and invasion of PCa cells (Gu et al., 2015). BAZ2‐ICR and GSK2801 were tested for their effect on PC3 (AR‐negative) and 22Rv1 (AR‐positive) PCa cells. In 2D‐cell culture, BAZ2‐ICR did not affect growth or viability, while GSK2801 significantly reduced growth rate at the highest concentrations tested without affecting viability (Figure 1a, b). This last effect could be ascribed to GSK2801 inhibition of BRD9 that, considering the reported *K*_D_, is significantly achieved only at concentrations ≥5 μM. At lower concentrations, the sole inhibition of BAZ2 proteins by GSK2801 does not affect cellular growth, in agreement with what observed using BAZ2‐ICR, which does not cross‐react with BRD9; this last protein has been reported to support proliferation in various cancer types, including PCa (Alpsoy et al., 2020; Dou et al., 2020). Nonetheless, the ability of BRD9 inhibitors to strongly induce cancer cells death has been disputed especially when compared with protein removal either by PROTACs or genetic knockdown (Remillard et al., 2017; Zoppi et al., 2019). Indeed, treatment with the BRD9‐specific inhibitor BI9564 only moderately affected 22Rv1 cell growth while being completely inactive on PC3 cells (Figure 1c, d). Co‐treatment with BAZ2‐ICR and BI9564 was not more effective than the single treatments (Figure 1e, f), demonstrating that the observed effects of GSK2801 on PCa cells growth cannot be ascribed to the simultaneous inhibition of BAZ2 and BRD9 bromodomains and configures probably as an off‐target effect. In agreement with the observed unaltered cellular growth, treatment with BAZ2‐ICR at saturating concentration (25 μM) did not comparably alter the transcription of a set of genes previously shown to be affected by BAZ2A genetic ablation, highlighting that the single interference with the bromodomain does not fully recapitulate the ablation phenotype (Figures S1, S2).

**FIGURE 2:**
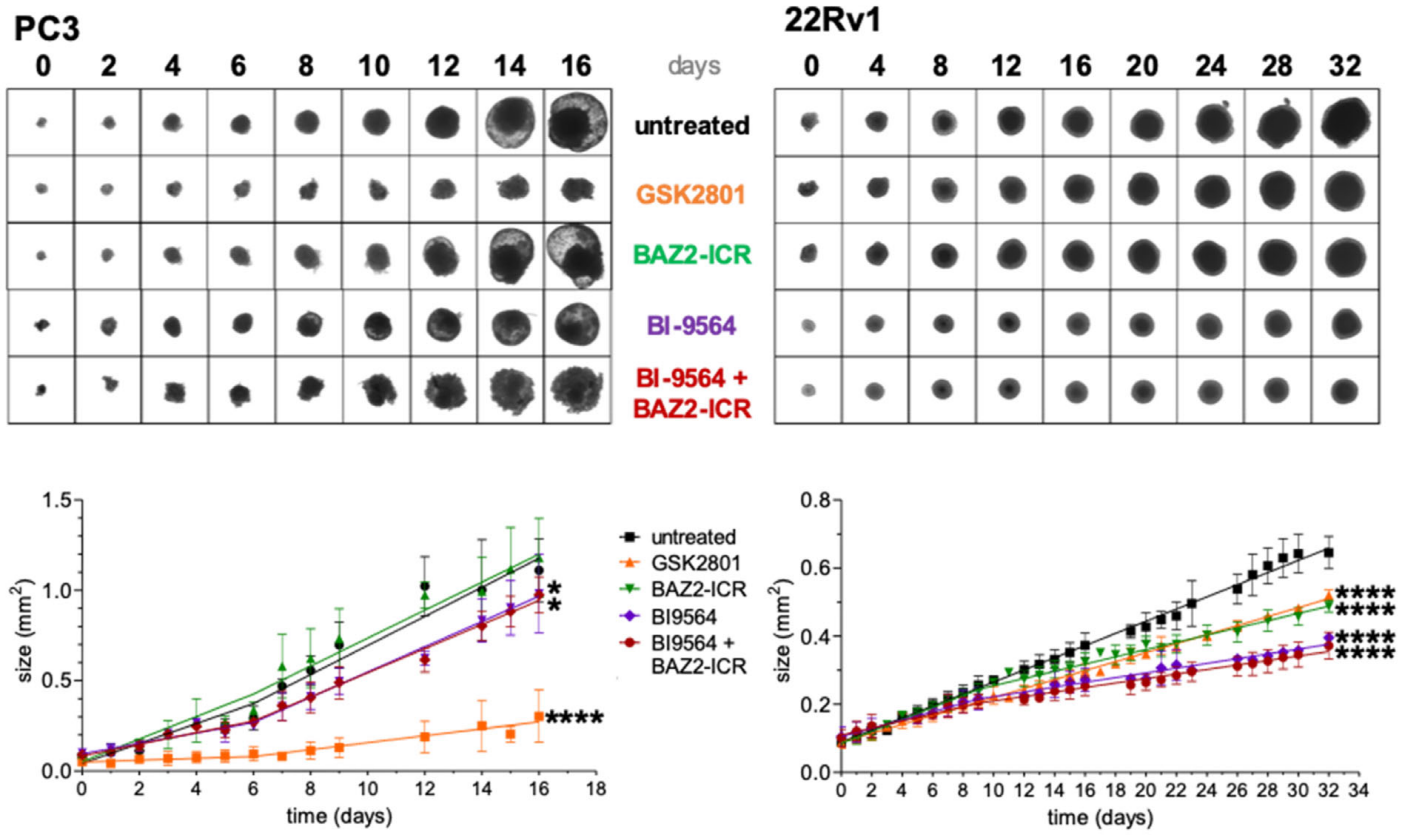
Effect of tested inhibitors on PCa spheroids. GSK2801 (25 μM) and BAZ2‐ICR (25 μM) similarly affect the growth of 22Rv1 cells in a 3D setting, while only GSK2801 is effective on PC3 cells. Co‐treatment with BAZ2‐ICR (25 μM) and BI9564 (20 μM) does not cause a different effect compared to treatment with BI9564 alone, nor reproduces the GSK2801 effects. Lines are obtained using the segmented linear regression analysis of the Graphpad Prism software. The growth rate of the spheroids changes at days 6 and 9 for PC3 and 22Rv1, respectively. The treatment was considered to have an effect when the slopes obtained from the lines of the treated condition were statistically different from the one of the untreated. ns = *p* > 0.05, *p < 0.05, ****p < 0.0001. The comparison was also made between BI9564 alone and co‐treatment with BI9564 and BAZ2‐ICR; slopes were not found to be statistically different for either cell line. For each replicate, 5/6 spheroids/conditions were seeded. Doubling time for the two cell lines is significantly different, about 24 and 50 h for PC3 and 22Rv1 cells, respectively.

GSK2801 slowed PC3 and 22Rv1 cellular growth also in a 3D setting. BAZ2‐ICR instead did not significantly affect PC3 spheroids, while interestingly impacting those deriving from 22Rv1 cells (Figure 2). In this last case, the spheroids maintain a regular and rounded shape differently from the untreated cells that grow larger and erratically. The observed effect reproduces in a different setting the recently reported efficacy of BAZ2‐ICR on PCa tumorspheres, but not on heterogeneous cell populations, deriving from its ability to impair cancer stem cells (Peña‐Hernández et al., 2021). BRD9 inhibition by BI9564 reduced the growth of 22Rv1 spheroids being less effective on PC3 in a similar setting (Figure 2). Co‐treatment with BI9564 and BAZ2‐ICR did not influence spheroids growth differently from the single treatment with BI9564, which exceeds and masks BAZ2‐ICR effects at the concentration tested. Comparison with the effects observed with the single GSK2801 treatment confirms the presence of confounding factors accompanying the use of the GSK inhibitor. Notably, GSK2801 was shown to strongly interferes with at least the melatonin receptor when screened on a panel of receptors, channels and transporters (https://www.thesgc.org/chemical-probes/GSK2801), differently in this respect from the clean BAZ2‐ICR profile (https://www.thesgc.org/chemical-probes/BAZ2-ICR).

The observed limited efficacy of BAZ2‐ICR, together with the suspected GSK2801 off‐target effect, could be overcome with the development of BAZ2A‐directed degraders. These would chemically phenocopy the genetic ablation effects as well as improve specificity looking for optimal surface complementarity between BAZ2A and ubiquitin ligases (Bondeson et al., 2018; Gadd et al., 2017; Smith et al., 2019). In this respect, crystallographic structures of BAZ2A in complex with chemical probes would drive the identification of growing vectors to move out of its pocket in different directions.

**FIGURE 3:**
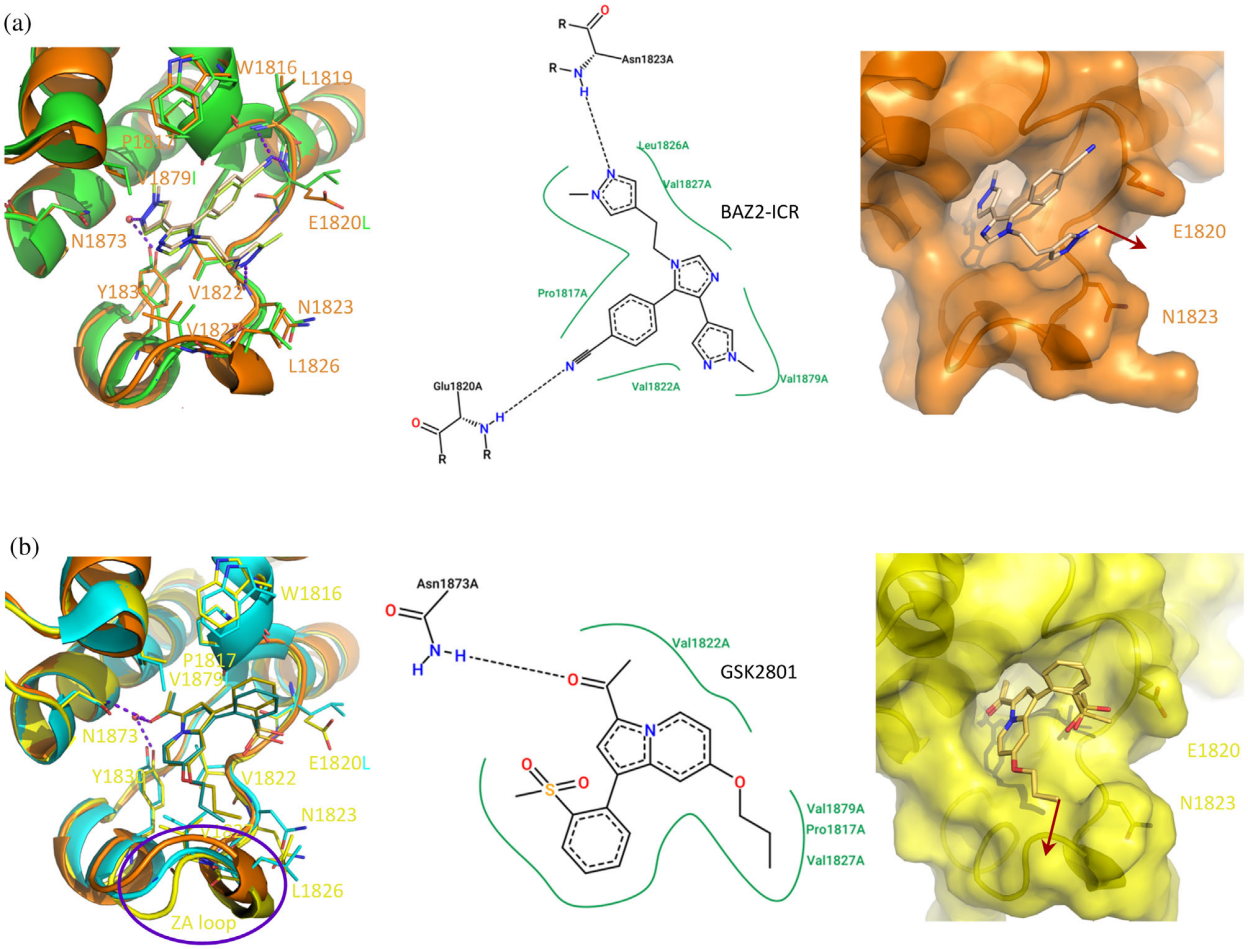
Crystallographic structures of BAZ2 chemical probes bound to BAZ2A. (a) Binding pose of BAZ2‐ICR (ivory) to BAZ2A (orange) superposed to BAZ2‐ICR (light green) bound to BAZ2B (green, PDB 4XUB). (b) GSK2801 (gold) in complex with BAZ2A (yellow) compared to the same inhibitor (turquoise) bound to BAZ2B (cyan, PDB 4RVR); the different conformations assumed by the ZA loop are circled in purple. Residues numbering refers to BAZ2A; substituted residues in BAZ2B are reported according to the structure color. Exit vectors for PROTACs generation are indicated with red arrows.

The co‐crystal structure of BAZ2A in complex with BAZ2‐ICR shows a binding pose almost superimposable to that previously reported for a BAZ2‐ICR analogue bound to BAZ2B (Figure 3a; Drouin et al., 2015). Slight tilting of the probe is imposed by the substitution of Ile1950 in BAZ2B with Val1879 in BAZ2A, which in turn is reflected in the benzonitrile ring being closer to the WPF (Trp‐Pro‐Phe) shelf in BAZ2A. The nitrile nitrogen is in contact with the main chain nitrogen of Glu1820 (at 2.9 Å, similarly to what observed in BAZ2B with main chain nitrogen of Leu1891 at 3.1 Å) but also with Leu1819 main chain nitrogen (3.1 Å in BAZ2A, 3.6 Å in BAZ2B). Interestingly, Glu1820 moves from the mm rotameric form, observed in the apo form (χ_1_ = −65°, χ_2_ = −58°), to the pt rotamer (χ_1_ = 63°, χ_2_ = −175°), folding on the side of BAZ2‐ICR and reducing its solvent accessible area. The Glu1820 carboxylic group is at about 4 Å distance from the probe benzonitrile and methyldiazole tails, these last interacting between them through π–π stacking. In the context of largely conserved interactions, the above comparison supports the similar affinity of the BAZ2‐ICR probe for BAZ2A and BAZ2B. The methyldiazole tail could be extended with a linker/E3 recruiter moving out of the pocket in the region delimited by Glu1820 and Asn1823 side chains.

GSK2801 interacts with BAZ2A slightly less efficiently than in BAZ2B (Chen et al., 2016). Binding poses are very similar in the two bromodomains as well as the observed interactions, with the notable exception of the hydrogen bond between a sulfonic oxygen and the Asn1894 main chain nitrogen (at 3.1 Å in BAZ2B). The same interaction is missing in BAZ2A, with the distance between the sulfone and Asn1823 increasing to 4.5 Å (Figure 3b). BAZ2A Glu1820 moves toward GSK2801 and closes the pocket, as observed with BAZ2‐ICR. Interestingly, the GSK2801 propoxyl tail induces the BAZ2A ZA loop to move slightly away from the Kac (acetylated lysine) pocket (with respect to what observed in the other BAZ2A structures), closely retracing the same loop in the BAZ2B‐GSK2801 structure. This configures as a very obvious extension point for the synthesis of a corresponding PROTACs, moving out of the BAZ2A pocket in a different direction with respect to BAZ2‐ICR.

To identify new BAZ2A binding scaffolds and chemical groups exploring its pocket differently from GSK2801 and BAZ2‐ICR, five additional chemical probes were selected as putative BAZ2 binders, together with the CBP/BAZ2 cross‐reactive inhibitor UP39. In particular, we were interested in positively‐charged tail groups for engaging in favorable electrostatic interactions with the negatively charged BAZ2A Glu1820, as a possible selectivity discriminant at the tip of the BAZ2A pocket (Table 1).

**TABLE 1:** Binding competition activities tested by AlphaScreen.

Compound	Structure	% residual binding BAZ2A (100 μM)	% residual binding BAZ2B (100 μM)	IC_50_ BAZ2A (μM)	IC_50_ BAZ2B (μM)	*K*_D_ BAZ2A (μM)
TP‐238	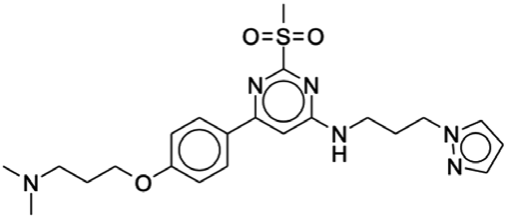	81 ± 3	77 ± 2	‐	‐	108 ± 6
GSK4027	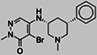	77 ± 5	70 ± 3	‐	‐	165 ± 8
GSK8814	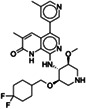	88 ± 3	86 ± 2	‐	‐	‐
SGC‐CBP30	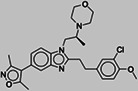	95 ± 4	92 ± 6	‐	‐	‐
I‐CBP112	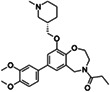	98 ± 2	96 ± 1	‐	‐	‐
UP39	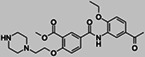	36 ± 5	42 ± 6	61 ± 4	73 ± 5	‐
UZH23	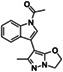	52 ± 9	62 ± 7	86 ± 9	> 100	‐
GSK2801	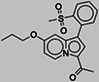	‐	‐	0.40 ± 0.03^a^	0.43 ± 0.02^a^	0.257^a^
BAZ2‐ICR	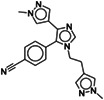	‐	‐	0.13^b^	0.18^b^	0.109^b^

^a^ In Reference Chen et al. (2016).

^b^ In Reference Drouin et al. (2015).

TP‐238 is a CECR2 binder (*K*_D_ = 10 nM), also active on BPTF and BRD9 with *K*_D_ = 120 nM and 1.1 μM, respectively, (Ycas et al., 2020). GSK4027 binds to the PCAF and GCN5 bromodomains with *K*_D_ = 1.4 nM for both, while also inhibiting other bromodomains with lower potency (BRPF1, BRPF3, BRD1, and BPTF in the range 100–150 nM) (Humphreys et al., 2017). GSK8814 is a potent nanomolar binder for ATAD2 bromodomains, interacting with lower affinity with TAF1 and TAF1L bromodomains (Bamborough et al., 2016). Finally, SGC‐CBP30 and I‐CBP112 are chemical probes for the CBP/EP300 bromodomains with weak off‐target activity on the first bromodomain of BRD4 (Hay et al., 2014; Picaud et al., 2015). In accordance with the selectivity profile reported previously, when tested on BAZ2 bromodomains, these five chemical probes showed limited activity, if any (Table 1). When screened by co‐crystallization, TP‐238, GSK4027, and UP39 were located in the BAZ2A Kac pocket, but not GSK8814, SGC‐CBP30, and I‐CBP112 confirming their inability to act as BAZ2A inhibitors.

**FIGURE 4:**
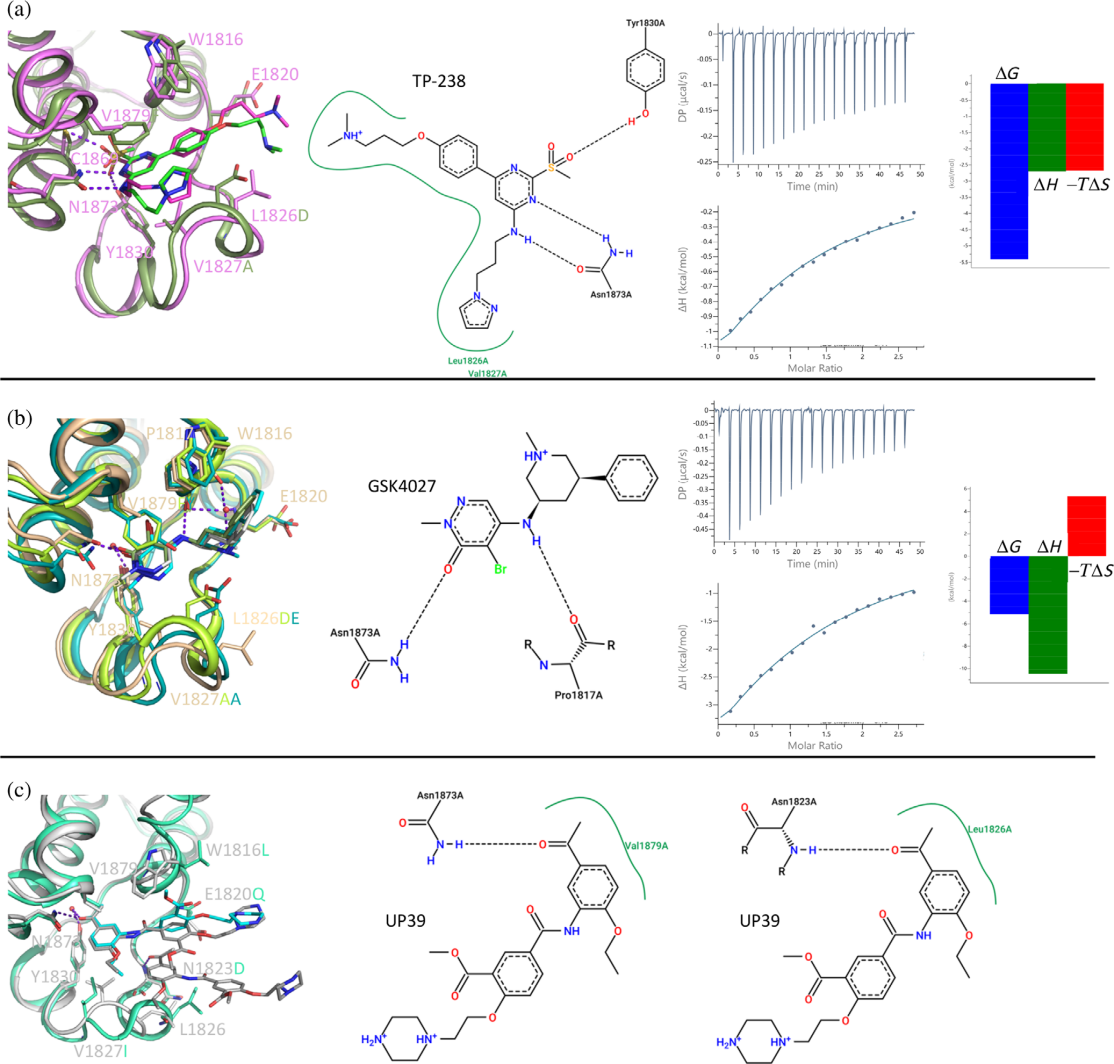
Crystallographic structures of TP‐238, GSK4027 and UP39 in complex with the BAZ2A bromodomain. Residues numbering refers to BAZ2A; substituted residues in BPTF, GCN5 and CBP are reported accordingly to the structure color. (a) Comparison of TP‐238 binding pose in BAZ2A (pink, TP‐238 in magenta) and BPTF (dark green, TP‐238 in green, PDB 7KDZ). ITC titration identifies limited but favorable enthalpic and entropic contributions (blank titration is shown as Figure S6). (b) GSK4027 binds to BAZ2A bromodomain (ivory, GSK4027 in white) similarly to BPTF (light green, GSK4027 in dark green, PDB 7K6R) and GCN5 (dark cyan, GSK4027 in cyan, PDB 5MLJ) bromodomains. The favorable enthalpic contribution is partially counteracted by the opposite entropic effect. (c) The largest divergence for compound UP39 bound to BAZ2A (white, UP39 in gray) or CBP (aquamarine, UP39 in cyan, PDB 5ENG) is the different orientation of the central methylbenzoate ring caused by the substitution of CBP Leu1109 with BAZ2A Trp1816.

TP‐238 binds similarly in BPTF and BAZ2A (Figure 4a). There are, however, numerous deviations in the interaction pattern due to the several sequence differences between the two bromodomains. First, the 2‐(methylsulfonyl)pyrimidin‐4‐amine headgroup binds slightly deeper in the Kac pocket of BAZ2A, displacing a structural water molecule, conserved in the vast majority of crystal structures of bromodomains in complex with small molecule ligands (Brand et al., 2015) and all complexes with peptides containing acetyl‐lysine (Marchand & Caflisch, 2015). Although the displacement of the structural water that bridges to the conserved tyrosine side chain (Tyr1830 in BAZ2A) has been observed for various bromodomains in complex with small molecules (Clegg et al., 2020; Crawford et al., 2016; Lolli & Caflisch, 2016), TP‐238 is the first ligand to achieve this displacement in BAZ2A. The sulfonyl group forms H‐bonds with side chains of Cys1869 and Tyr1830, while the aminopyrimidine faces Asn1873, generating two additional H‐bonds. In BPTF, the structural water molecule is not displaced and bridges the TP‐238 sulfonyl group to Tyr2964 side chain, as typically seen in most of the bromodomain/inhibitor complexes. An additional H‐bond is formed between the sulfonyl group and Asn3007, while the methyl group occupies the hydrophobic cavity reserved for it, as observed in complex with Kac peptides. The aminopyrimidine interactions with Asn3007 are substantially identical to what described for BAZ2A. Moreover, the BPTF gatekeeper residue Phe3013, stacking with the pyrimidine ring, is substituted by the more protruding Val1879 in BAZ2A. This, together with the substitution Ala2961 to Val1827, imposes to TP‐238 to locate in a slightly different plane in the two bromodomains. The propyl‐pyrazole tail is located similarly; however, it shows different T‐shaped stacking interactions: to Phe3013 in BPTF, while intramolecularly to the TP‐238 phenyl substituent in BAZ2A. Greater divergence is observed for the dimethylamino‐propoxy‐phenyl tail. The phenyl group stacks in both cases to the Trp residue of the WPF shelf, but the terminal charged amino group is contacting Asp2960 in BPTF (Leu1826 in BAZ2A), while largely exposed to the solvent in BAZ2A and >5 Å away from Glu1820. Overall, TP‐238 candidates as an interesting molecule whose pliability can be explored to develop bromodomain inhibitors with different selectivity profiles. In particular, we notice that the achieved displacement of the structural water molecule in BAZ2A brings the inhibitor close to Cys1869 side chain, suggesting a possible evolution toward a covalent inhibitor. Further optimization of the dimethylamino tail could be envisaged by either reaching Glu1820 or changing it to a hydrophobic group contacting Leu1826.

Similar considerations are valid for GSK4027, whose structure in complex with BAZ2A is presented here and compared with those available in complex with GCN5 and BPTF (Figure 4b). The different gatekeeper residues (Tyr814 in GCN5, Phe3013 in BPTF, and Val1879 in BAZ2A), together with the substitution of GCN5 Ala762 or Ala2961 in BPTF with BAZ2A Val1827, causes the 5‐amino‐4‐bromo‐2‐methyl‐3‐pyridazinone headgroup to tilt differently. Main polar interactions are anyway conserved, that is, those between the carbonyl oxygen and Tyr1830/Asn1873, and between the bromine atom and amino group from the GSK4027 headgroup and Pro1817 main chain oxygen. The phenyl‐piperidine tail locates similarly in the three bromodomains with optimal interactions with the conserved WPF shelf. However, the charged piperidine nitrogen is involved in a strong salt bridge with GCN5 Glu761 at 2.7 Å or BPTF Asp2960 at 3.1 Å (Leu1826 in BAZ2A), while failing to interact with BAZ2A Glu1820. Interestingly, in BAZ2A structure a water molecule is tetrahedrally coordinated by the piperidine nitrogen, Glu1820 main chain nitrogen and main chain oxygens from Trp1816 and Pro1817. Although the aimed interaction with Glu1820 side chain is missing, the BAZ2A/GSK4027 complex provides a Kac mimic never observed in complex with BAZ2A, and a new anchoring point with main chain atoms of BAZ2A. Exploitation of this scaffold would however require a de novo hit optimization campaign toward BAZ2A, possibly extending the N‐methyl of the piperidine ring with groups able to intercept interactions with Glu1820 side chain or with the protein matrix (by replacing the bridging water molecule and relieving the entropic penalty, see ITC below).

ITC confirmed that both TP‐238 and GSK4027 are poor BAZ2A binders with *K*_D_ in the very high micromolar range (Figure 4a, b, Table 2). It is worth noticing however that these values may be slightly overestimated. The presence of 0.25% DMSO in the ITC experiments leads to less favorable *K*_D_ values as DMSO competes for binding to the conserved Asn (Lolli & Battistutta, 2013; Navratilova et al., 2016). Interestingly however, the entropic change associated with their binding is opposite, favorable in case of TP‐238 and penalizing for GSK4027. This agrees with the observed release of water molecules from the pocket to the bulk solvent operated by TP‐238 and with the opposite freezing of solvent molecules observed in the GSK4027 structure. Notably, the thermodynamic fingerprints of GSK2801 and BAZ2‐ICR binding to BAZ2 bromodomains and TP‐238 binding to BPTF and CECR2 bromodomains (https://www.thesgc.org/chemical-probes/epigenetics) are relatively more similar to GSK4027; these interactions, not displacing any of the structural water molecules, are also characterized by an adverse entropic contribution in the context of a much more favorable enthalpic change, as also seen for the vast majority of bromodomain inhibitors. Indeed, the change in sign of the entropic term observed for TP‐238 binding to BAZ2A (compared to BPTF and CECR2) closely resembles the effect reported for crotonyllysine binding to the TAF1 bromodomain (compared to Kac), also displacing an additional water molecule from the binding pocket (Flynn et al., 2015).

**TABLE 2:** Thermodynamic binding parameters.

	Δ*H* (kcal/Mol)	*T*Δ*S* (kcal/mol)	Δ*G* (kcal/mol)	*N* (sites)	*K*_D_ BAZ2A (μM)
TP‐238	−2.73 ± 0.07	2.68	−5.41	1	108 ± 6
GSK4027	−11.0 ± 0.3	−5.8	−5.2	1	165 ± 8

Compound UP39 was developed as a CBP bromodomain inhibitor with a *K*_D_ = 1.4 μM (Zhu et al., 2018). In BAZ2A, the acetylbenzene headgroup binds very similarly to what reported for CBP, with hydrogen bonds to Asn1873 and Tyr1830 (the last being water‐bridged) and the gatekeeper valine conserved in the two bromodomains (Figure 4c). The long tail, although pointing in the same direction observed in CBP, assumes a different pose. The central methylbenzoate ring is tilted of about 70°, due to the substitution of Leu1109 in CBP with Trp1816 in BAZ2A. Overall, an almost coplanar organization of the UP39 ring systems is observed in BAZ2A, with a T‐shaped stacking interaction between the methylbenzoate ring and Trp1816. Glu1820 assumes the pt rotameric orientation (χ_1_ = 63°, χ_2_ = −175°) moving toward the terminal positively‐charged UP39 piperazine ring, however at a distance >5 Å. A second UP39 molecule is bound to the BAZ2A bromodomain. The acetylbenzene headgroup drives the interaction with a hydrogen bond between the acetyl oxygen and the Asn1823 main chain nitrogen and through the stacking with the central methylbenzoate ring of the other UP39 molecule. This generates a four‐layer sandwich, similar to that observed in the complex with BAZ2‐ICR, with the two UP39 molecules stacked in between side chains of Trp1816 and Leu1826. The tail of the second UP39 molecule protrudes away from the bromodomain and is stabilized by additional interactions with a BAZ2A symmetry‐related copy. This four‐layer organization is also observed in the different CBP/BAZ2 cross‐reactive inhibitor UZH23, described in the supplementary material (Figure S4), and in additional BAZ2A hit compounds recently identified (Dalle Vedove et al., 2021, 2022). These last advantageously exploit both self‐stacking and H‐bond to Asn1823, which could be achieved in UP39 or UZH23 derivatives.

## 3 CONCLUSIONS

This work first demonstrates that the activities of GSK2801 and BAZ2‐ICR are not superimposable and the effects associated with the use of GSK2801 do not appear to derive from the sole interference with BAZ2 and BRD9 bromodomains. We then show that the sole interference with the BAZ2A bromodomain by BAZ2‐ICR does not reproduce the effects observed by its genetic ablation. BAZ2 chemical probes could however be evolved in PROTACs to improve their cytostatic and anti‐metastatic efficacies; the crystallographic structures here disclosed identify suitable derivatization points to move out of the BAZ2A Kac pocket allowing to present BAZ2A differently to E3 ligases and increasing the probability of obtaining effective degraders. Finally, structures of BAZ2A in complex with TP‐238, GSK4027 and UP39 provide additional headgroup and peculiar binding modes for the development of new BAZ2 inhibitors.

## 4 METHODS

BAZ2A and BAZ2B bromodomains were produced and their activity evaluated by AlphaScreen as previously reported (Dalle Vedove et al., 2018; Spiliotopoulos et al., 2017). BAZ2A bromodomain was crystallized as detailed in an earlier work (Spiliotopoulos et al., 2017). Diffraction data were collected at the Elettra Synchrotron Light Source (Trieste, Italy), XRD1 and XRD2 beamlines, and the European Synchrotron Radiation Facility (ESRF, Grenoble, France), ID30A‐1/MASSIF‐1 beamline. Data were processed and structures were solved as described elsewhere (Dalle Vedove et al., 2018). Data collection and refinement statistics are reported in Table S1. Electron densities for the bound inhibitors are shown in Figure S5. Chemical synthesis of compounds UP39 and UZH23 has already been reported (Unzue et al., 2016; Zhu et al., 2018). A detailed description of cell culture methods and ITC protocol is provided in the Supporting Information.

## AUTHOR CONTRIBUTIONS

**Giulia Cazzanelli:** Conceptualization; Investigation; Writing ‐ review & editing; Formal analysis; Methodology. **Andrea Dalle Vedove:** Investigation; Formal analysis; Methodology. **Eleonora Parolin:** Investigation. **Vito Giuseppe D'Agostino:** Investigation; Formal analysis. **Andrea Unzue:** Resources. **Cristina Nevado:** Resources. **Amedeo Caflisch:** Conceptualization; Writing ‐ review & editing; Supervision; Funding acquisition; Resources. **Graziano Lolli:** Conceptualization; Writing ‐ original draft; Writing ‐ review & editing; Formal analysis; Supervision; Funding acquisition; Resources.

## CONFLICT OF INTEREST STATEMENT

The authors declare no competing financial interest.

## Supporting information


**Data S1:** Supporting Information
