# Supplementary material for: Reevaluation of bromodomain ligands targeting BAZ2A
Source: Protein Sci. 2023 Sep 1;32(9):e4752. doi: 10.1002/pro.4752 (PMC10464297; doi:10.1002/pro.4752)
Supplement: Supplementary file 1 — Data S1: Supporting Information [file PRO-32-e4752-s001.pdf]

SUPPLEMENTARY MATERIAL

Reevaluation of bromodomain ligands targeting BAZ2A

Giulia Cazzanelli[1], Andrea Dalle Vedove[1], Eleonora Parolin[1], Vito Giuseppe D'Agostino[1], Andrea Unzue[2], Cristina Nevado[2], Amedeo Caflisch[3] and Graziano Lolli[1*]

Author Affiliations:

[1]Department of Cellular, Computational and Integrative Biology - CIBio, University of Trento, via Sommarive 9, 38123 Povo - Trento, Italy

[2]Department of Chemistry, University of Zürich, Winterthurerstrasse 190, CH-8057 Zürich, Switzerland

[3]Department of Biochemistry, University of Zürich, Winterthurerstrasse 190, CH-8057 Zürich, Switzerland

**Correspondence to Graziano Lolli*: graziano.lolli@unitn.it.

SUPPLEMENTARY RESULTS

BAZ2A-UZH23 crystal structure

A similar stacking is observed for compound UZH23 (CBP K_d = 6.2 μM [35]), again with two molecules in the BAZ2A Kac pocket (Suppl. Fig. 4). The first one forms the usual hydrogen bonds between the acetylindole moiety and the Tyr1830-Asn1873 couple. The pyrazole-oxazole moiety runs perpendicular to Trp1816 with a 34° tilt with respect to the indole ring; a water molecule bridges one of its nitrogen to Glu1820. The second UZH23 is again in hydrogen bond contact with Asn1823 main chain nitrogen with its indole ring sandwiched between the pyrazole-oxazole ring of the first UZH23 molecule and Leu1826.

Notably, UZH23 and GSK2801 share an almost identical headgroup while having different selectivity. While GSK2801 is a potent BAZ2 inhibitor selective against CBP, UZH23 binds CBP only marginally better than BAZ2 bromodomains. Further exploration of these scaffolds could reveal successful for the development of a dual BAZ2/CBP inhibitor.

SUPPLEMENTARY METHODS

Cell cultures, proliferation assay and spheroid growth

PC3 and 22Rv1 cells were cultured in RPMI 1640 medium containing 10% FBS, 1% penicillin-streptomycin and 2 mM L-glutamine. All cells were regularly tested for mycoplasma contamination.

For the proliferation assay, cells were counted with a hemocytometer and seeded in triplicates in 96-well plates (4.5x10^3 cells/well for 22Rv1 and 2.0x10^3 for PC3) and let adhere overnight at 37°C, 5% CO_2. The following day, the medium was replaced with RPMI 1640 containing either the carrier (0,1% DMSO) or the inhibitors (0.5 – 5.0 – 10.0 – 25.0 μM BAZ2-ICR; 0.5 – 2.5 – 5.0 – 10.0 – 25.0 μM GSK2801; 0.1 – 0.25 – 0.5 – 1.0 – 2.5 – 5.0 – 10.0 – 20.0 μM). Alternatively, the cells were transfected with siRNA for BAZ2A, RLUC or KIF11, as detailed below. The cells were incubated for 1, 3, 6 (PC3) and 9 days (22Rv1) and the medium containing either the carrier or the inhibitors was replaced every 3 days. Cell proliferation and viability were determined at day 1, 3, 6 and 9 by adding 10% resazurin sodium salt solution (0.03 mg/ml powder from Sigma-Aldrich) directly into each well and incubating them for 4h at 37°C, 5% CO_2. The absorbance at 570/600 nm was measured using a Varioskan plate reader. The reduction of the resazurin sodium salt was calculated using the formula: (Abs x – Abs 0.1%DMSO day1)/(Abs 0.1%DMSO day9 - Abs 0.1%DMSO day1). The growth rate was analysed using the linear regression algorithm of GraphPad Prism. The appropriateness of the model was evaluated by considering the R square values and the Runs test (Suppl. Table 3-5).

Spheroids were obtained by plating PC3 and 22Rv1 cells in low-adhesion round bottom 96-well plates. For 22Rv1, 2500 cells/well were seeded in 200 μl RPMI 1640; for PC3, 250 cells/well were seeded in 200 μl RPMI 1640 supplemented with 1.5% Matrigel. After seeding, the cells were centrifuged for 10 min at 1200 x g at 4°C. Spheroids were given 48 hours to form after seeding before adding the treatment or the carrier (0.1% DMSO). Medium supplemented with treatment/carrier was changed every 48 hours, for a total of 32 days for 22Rv1 and 16 days for PC3. Each spheroid was imaged in brightfield every day or every other day (after day 20), using a Leica DM IL microscope

equipped with a Leica DFC450C digital camera. The area of each spheroid was measured using ImageJ and the average area for each condition was calculated. The growth rate was analysed using the segmental linear regression algorithm of GraphPad Prism. The appropriateness of the model was evaluated by considering the R square values and the Replicates test (Suppl. Table 6).

siRNA transfection

Cells were counted and plated in 6 well (for RNA extraction) or 96 well (for growth curve) at a fixed amount ($6.0x10^4$ cells/well for PC3; $1.35x10^5$ cells/well for 22Rv1 in 6 well; $2.0x10^3$ for PC3 and $4.5x10^3$ cells/well for 22Rv1) and let attach overnight at 37°C, 5% CO_2. They were then transfected with either 50 nM esiRNA BAZ2A, esiRNA RLUC (negative control) or esiRNA KIF11 (positive control) using JetPRIME ® reagent, according to manufacturer's instruction. esiRNA (endoribonuclease-prepared siRNA, Sigma-Aldrich), are pools of siRNA that all target the same mRNA sequence, ensuring minimal risk of off-target effects. After 3 days, the cells were treated again with the same amount of esiRNA. The gene expression was analyzed after 6 days from the cells seeded in the 6 well, while the viability was analyzed in the same way as for the cells treated with the inhibitors.

RT-qPCR

The total RNA content was extracted from cells treated for 6 days with 25 μM BAZ2-ICR or with the carrier and from cells treated with 50 nM siRNA against BAZA (MISSION® esiRNA, Merck). Cells were previously counted and plated in 6 well at a fixed amount (60.000 cells/well for PC3; 135.000 cells/well for 22Rv1). The RNA was extracted using the phenol-chloroform method and its concentration was measured by Nano spectrophotometer. 1 μg of RNA per sample was retrotranscribed using the High-Capacity cDNA Reverse Transcription Kit (Applied Biosystems) according to the manufacturer's instructions. The obtained cDNA was diluted 1:8 and used for RT-qPCR, performed on a Biorad CFX96 thermocycler using the Excel-Taq FAST qPCR SybrGreen

(Smobio) reagent, according to the manufacturer's instructions. Every reaction was performed in triplicate and accompanied by the two negative controls consisting of water or non-retrotranscribed RNA as template. The primers used are listed in Suppl. Table 7. Genes expression was normalized using the Pfaffl method with three different housekeeping genes.

Western blot

Total protein content was extracted from cells transfected with siRNA BAZ2A or siRNA RLUC (negative control). Cells were seeded as for RT-qPCR experiment. Cells were lysed using RIPA buffer and protein concentration was determined by Bradford Reagent (Sigma-Aldrich), according to the manufacturer's instructions. 25 μg of proteins per sample in Laemmli Buffer 4x were run in a 6% polyacrylamide gel until complete separation. The proteins were then blotted on a PDVF (polyvinylidene difluoride) membrane, which was blocked in 5% skimmed milk and 0.1% Tween 20 in TBS for 2h at room temperature and then treated with the appropriate primary (1:1000 anti-BAZ2A Abcam ab290639 or 1:4000 anti-α-actinin Santa Cruz Biotechnology sc-17829; 4°C overnight) and secondary (1:10.000 goat anti-mouse HRP Invitrogen 62-6520 or 1:10.000 goat anti-rabbit HRP Cohesion Biosciences CSA2115; 2h at room temperature) antibodies and revealed by chemiluminescence (ECL Bio-Rad).

Isothermal Titration Calorimetry

ITC experiments were performed using an MicroCal PEAQ-ITC microcalorimeter (Malvern Panalytical, UK) at 25°C with a stirring speed of 750 rpm in 20mM Tris pH 7.8, 0.2 M NaCl, 0.2 mM TCEP and 0.25% DMSO. For each experiment, one 0.4 μl injection followed by eighteen 2 μl injections have been performed, with 150 seconds separating each injection. The inhibitors were tested at 70 μM in the cell with His-Baz2A in the syringe at 1 mM. A blank run with only the buffer was carried out (Suppl. Fig. S6). Data analysis was done with PEAQ-ITC Analysis software, fitting the curve with the 'one site' model.

Model quality

Mogul analysis (available in the PDB validation reports for all structures at rcsb.org) shows some ligand geometry distortions. Those distortions are not forced by the protein-ligand interactions, but derive from the refinement procedure that was applied without imposing large values for the geometrical restraint weight. GSK4027 geometry is accompanied by comparatively larger RMSZ values (Table S1). Indeed, GSK4027 has a partial occupancy (0.72), and its electron density is altered by that deriving from water molecules bound in the apo fraction. The larger distortion of GSK4027 compared to the other inhibitors derives from the refinement software trying to adapt the GSK4027 structure in this partially blurred and spurious density.

Following the observation from one of the assigned Reviewers that diffraction data are affected by significant anisotropy, we exploited the STARANISO server (https://staraniso.globalphasing.org/cgi-bin/staraniso.cgi) to optimize data reduction and structure refinement. In particular, regarding structures 7BL8, 7BLB, 7BLC and 7BLD, they refine worse when the default STARANISO files are used (max resolution 0.3-0.5 Å higher than for the deposited structures, depending on the degree of anisotropy with spherical completeness about 10% in all cases and $CC^{1/2}$ 0.5 or slightly higher). Statistics improve when resolution is cut at spherical completeness > 50%. This is generally achieved at a resolution 0.2-0.3 Å higher than the deposited structures, with an improvement in the range 0.4-0.7 for both Rwork and Rfree. Nonetheless, electron density maps did not improve significantly, especially regarding the ligands; their poses (or those of surrounding residues) are not affected, nor do additional features or ambiguities appear.

Table S1. Data Collection and Refinement Statistics

	BAZ2-ICR	GSK2801	TP-238	GSK4027	UP39	UZH23
Data Collection						
Beamline	ESRF-ID30A-1	Elettra-XRD2	Elettra-XRD2	Elettra-XRD2	ESRF-ID30A-1	Elettra-XRD1
Space group	$P3_121$	$C222_1$	$P2_12_12_1$	$P3_121$	$P3_121$	$P3_121$
Unit-cell parameters (Å, °)	a = 95.32<br>b = 95.32<br>c = 32.88	a = 44.27<br>b = 56.80<br>c = 76.62	a = 35.65<br>b = 50.94<br>c = 67.73	a = 94.09<br>b = 94.09<br>c = 33.34	a = 94.95<br>b = 94.95<br>c = 32.35	a = 94.87<br>b = 94.87<br>c = 32.54
Wavelength (Å)	0.966	0.943	1.000	1.000	0.966	1.000
Resolution (Å)	47.66-2.50 (2.60-2.50)	38.31-1.30 (1.32-1.30)	40.71-1.09 (1.11-1.09)	47.04-2.30 (2.38-2.30)	82.23-2.30 (2.38-2.30)	47.44-2.35 (2.43-2.35)
R_{merge} (%)	10.9 (48.3)	5.4 (115.0)	5.5 (66.2)	18.4 (119.1)	21.6 (155.5)	24.8 (161.4)
R_{meas} (%)	12.9 (56.7)	5.7 (119.9)	5.7 (69.8)	18.9 (123.5)	22.8 (163.4)	26.0 (169.1)
R_{pim} (%)	6.8 (29.4)	1.6 (33.6)	1.6 (21.9)	4.5 (32.0)	7.2 (49.7)	7.8 (49.9)
$<I/\sigma(I)>$	7.7 (1.8)	23.0 (2.3)	21.9 (3.0)	11.5 (2.3)	8.5 (1.8)	9.8 (1.9)
$CC^{1/2}$	0.988 (0.797)	1.000 (0.839)	0.999 (0.892)	0.997 (0.858)	0.992 (0.760)	0.994 (0.816)
Completeness (%)	98.7 (97.9)	100.0 (100.0)	98.3 (95.2)	100.0 (100.0)	95.5 (100.0)	100.0 (100.0)
Multiplicity	3.4 (3.5)	12.4 (12.6)	12.1 (9.8)	17.3 (14.3)	9.9 (10.6)	10.8 (11.3)
Refinement						
Resolution (Å)	47.66-2.50	38.31-1.30	40.71-1.09	47.04-2.30	47.49-2.30	31.06-2.35
R_{work}/R_{free} (%)	20.3/23.9	15.6/18.0	13.9/16.1	20.0-22.6	19.7/21.8	19.0/23.0
Total number of atoms/mean B-factor (Å^2)	937/42.1	1940/23.9	2054/20.7	937/43.6	959/51.9	955/40.3
Protein number of atoms/mean B-factor (Å^2)	861/42.8	1727/23.6	1841/19.3	852/43.6	843/51.5	843/40.4
Water molecules/mean B-factor (Å^2)	49/37.3	109/29.4	151/30.5	62/42.4	48/41.4	70/39.7
Ligands number of atoms/mean B-factor (Å^2)	27/27.4	104/17.2	62/15.1	23/45.0	68/64.7	42/40.3
Ramachandran outliers	0	0	0	0	0	0
Ramachandran Z-score*	-2.28 ± 0.80	0.85 ± 0.89	1.99 ± 0.87	-2.70 ± 0.70	-2.25 ± 0.91	-0.55 ± 0.89
RMSZ bond lengths (protein)#	0.42	0.43	0.38	0.43	0.41	0.39
RMSZ bond angles (protein)#	0.53	0.62	0.58	0.54	0.53	0.52
RMSZ bond lengths (ligands)§	1.60	1.22	1.01	4.52	2.21	1.82
RMSZ bond angles (ligands)§	1.48	1.66	1.26	2.26	1.19	1.72
R.m.s. deviations						
Bond lengths (Å)	0.008	0.008	0.008	0.007	0.007	0.007
Bond angles (°)	0.86	1.03	1.10	0.88	0.95	0.80
PDB entry	7BL8	7BL9	7BLA	7BLB	7BLC	7BLD

* Sobolev O.V., Afonine P.V., Moriarty N.W., Hekkelman M.L., Joosten R.P., Perrakis A., Adams P.D. (2020) A Global Ramachandran Score Identifies Protein Structures with Unlikely Stereochemistry. *Structure 28*, 1249-1258.e2.

# Williams C.J., Headd J.J., Moriarty N.W., Prisant M.G., Videau L.L., Deis L.N., Verma V., Keedy D.A., Hintze B.J., Chen V.B., Jain S., Lewis S.M., Arendall W.B. 3rd, Snoeyink J., Adams P.D., Lovell S.C., Richardson J.S., Richardson D.C. (2018) MolProbity: More and better reference data for improved all-atom structure validation. *Protein Sci. 27*, 293-315.

§ Cottrell S.J., Olsson T.S., Taylor R., Cole J.C., Liebeschuetz J.W. (2012) Validating and understanding ring conformations using small molecule crystallographic data. *J. Chem. Inf. Model. 52,* 956-62

Table S2. Analysis of fitness of the linear regression model for the growth curve of PC3 and 22Rv1 treated with increasing concentration of BAZ2-ICR and GSK2801.

		**BAZ2-ICR (µM)**				**GSK801 (µM)**				
	**untreated**	**0,5**	**5**	**10**	**25**	**0,5**	**2,5**	**5**	**10**	**25**
**PC3**										
Best-fit values										
Slope	0,1585 ± 0,003949	0,1625 ± 0,004070	0,1599 ± 0,005100	0,1532 ± 0,008860	0,1580 ± 0,003917	0,1514 ± 0,003870	0,1455 ± 0,007743	0,1396 ± 0,01062	0,1068 ± 0,007205	0,08390 ± 0,009332
Goodness of Fit										
R square	0,994	0,994	0,990	0,968	0,994	0,994	0,973	0,945	0,957	0,861
Sy.x	0,031	0,032	0,040	0,070	0,031	0,031	0,061	0,084	0,057	0,077
Is slope significantly non-zero?										
F	1612	1595	982,9	298,9	1627	1530	353,3	172,7	219,7	80,81
DFn, DFd	1,000, 10,00	1,000, 10,00	1,000, 10,00	1,000, 10,00	1,000, 10,00	1,000, 10,00	1,000, 10,00	1,000, 10,00	1,000, 10,00	1,000, 13,00
P value	< 0,0001	< 0,0001	< 0,0001	< 0,0001	< 0,0001	< 0,0001	< 0,0001	< 0,0001	< 0,0001	< 0,0001
Deviation from zero?	Significant	Significant	Significant	Significant	Significant	Significant	Significant	Significant	Significant	Significant
Runs test										
Points above line	1	2	2	1	1	1	1	1	2	1
Points below line	3	2	2	3	3	3	3	3	2	3
Number of runs	3	4	4	3	3	3	3	3	4	3
P value (runs test)	1,00	1,00	1,00	1,00	1,00	1,00	1,00	1,00	1,00	1,00
Deviation from linearity	Not Significant	Not Significant	Not Significant	Not Significant	Not Significant	Not Significant	Not Significant	Not Significant	Not Significant	Not Significant
**22Rv1**										
Best-fit values										
Slope	0,1071 ± 0,003080	0,1041 ± 0,004429	0,1046 ± 0,003266	0,1034 ± 0,003959	0,1066 ± 0,003891	0,1090 ± 0,003671	0,1006 ± 0,003759	0,08978 ± 0,003220	0,07420 ± 0,003343	0,03702 ± 0,004161
Goodness of Fit										
R square	0,989	0,977	0,987	0,981	0,983	0,985	0,982	0,984	0,974	0,859
Sy.x	0,03949	0,05678	0,04187	0,05076	0,04989	0,04707	0,0482	0,04128	0,04287	0,05335
Is slope significantly non-zero?										
F	1209	552,3	1026	681,9	751,3	881,6	716,5	777,6	492,5	79,15
DFn, DFd	1,000, 13,00	1,000, 13,00	1,000, 13,00	1,000, 13,00	1,000, 13,00	1,000, 13,00	1,000, 13,00	1,000, 13,00	1,000, 13,00	1,000, 13,00
P value	< 0,0001	< 0,0001	< 0,0001	< 0,0001	< 0,0001	< 0,0001	< 0,0001	< 0,0001	< 0,0001	< 0,0001
Deviation from zero?	Significant	Significant	Significant	Significant	Significant	Significant	Significant	Significant	Significant	Significant
Runs test										
Points above line	2	3	3	3	2	3	3	2	3	3
Points below line	3	2	2	2	3	2	2	3	2	2
Number of runs	5	3	3	3	5	3	3	3	4	4
P value (runs test)	1,00	0,50	0,50	0,50	1,00	0,50	0,50	0,50	0,90	0,90
Deviation from linearity	Not Significant	Not Significant	Not Significant	Not Significant	Not Significant	Not Significant	Not Significant	Not Significant	Not Significant	Not Significant

Table S3. Analysis of fitness of the linear regression model for the growth curve of PC3 and 22Rv1 treated with increasing concentration of BI9564 alone and in combination with 25 µM BAZ2-ICR.

		**BI9564 (µM)**							
	**untreated**	**0,1**	**0,25**	**0,5**	**1**	**2,5**	**5**	**10**	**20**
**PC3**									
Best-fit values									
Slope	0,1572 ± 0,01069	0,1514 ± 0,01172	0,1530 ± 0,01046	0,1594 ± 0,01019	0,1557 ± 0,01027	0,1485 ± 0,01035	0,1453 ± 0,008944	0,1454 ± 0,009303	0,1470 ± 0,007297
Is slope significantly non-zero?									
F	216,3	166,9	214,2	244,8	229,8	205,8	264	244,2	406,1
DFn, DFd	1,000, 18,00	1,000, 18,00	1,000, 18,00	1,000, 18,00	1,000, 18,00	1,000, 18,00	1,000, 18,00	1,000, 18,00	1,000, 18,00
P value	< 0,0001	< 0,0001	< 0,0001	< 0,0001	< 0,0001	< 0,0001	< 0,0001	< 0,0001	< 0,0001
Deviation from zero?	Significant	Significant	Significant	Significant	Significant	Significant	Significant	Significant	Significant
Runs test									
Points above line	1	1	1	1	1	1	1	1	1
Points below line	3	3	3	3	3	3	3	3	3
Number of runs	3	3	3	3	3	3	3	3	3
P value (runs test)	1,00	1,00	1,00	1,00	1,00	1,00	1,00	1,00	1,00
Deviation from linearity	Not Significant	Not Significant	Not Significant	Not Significant	Not Significant	Not Significant	Not Significant	Not Significant	Not Significant
		**BI9564 (µM) + 25 µM BAZ2-ICR**							
Best-fit values									
Slope	0,1572 ± 0,01069	0,1514 ± 0,006086	0,1494 ± 0,005543	0,1489 ± 0,006893	0,1440 ± 0,006007	0,1494 ± 0,005377	0,1380 ± 0,005944	0,1360 ± 0,005935	0,1370 ± 0,006528
Goodness of Fit									
R square	0,9232	0,9794	0,9824	0,9729	0,9779	0,9835	0,9764	0,9758	0,9713
Sy.x	0,1095	0,05126	0,04668	0,05806	0,05059	0,04528	0,05006	0,04998	0,05498
Is slope significantly non-zero?									
F	216,3	618,5	726,5	466,3	574,4	772,6	538,9	524,9	440,2
DFn, DFd	1,000, 18,00	1,000, 13,00	1,000, 13,00	1,000, 13,00	1,000, 13,00	1,000, 13,00	1,000, 13,00	1,000, 13,00	1,000, 13,00
P value	< 0,0001	< 0,0001	< 0,0001	< 0,0001	< 0,0001	< 0,0001	< 0,0001	< 0,0001	< 0,0001
Deviation from zero?	Significant	Significant	Significant	Significant	Significant	Significant	Significant	Significant	Significant
Runs test									
Points above line	1	1	1	1	1	1	1	1	2
Points below line	3	3	3	3	3	3	3	3	2
Number of runs	3	3	3	3	3	3	3	3	3
P value (runs test)	1,00	1,00	1,00	1,00	1,00	1,00	1,00	1,00	0,67
Deviation from linearity	Not Significant	Not Significant	Not Significant	Not Significant	Not Significant	Not Significant	Not Significant	Not Significant	Not Significant
		**BI9564 (µM)**							
**22Rv1**									
Best-fit values									
Slope	0,1048 ± 0,004124	0,1043 ± 0,002976	0,1062 ± 0,003306	0,1057 ± 0,002879	0,1046 ± 0,003146	0,09943 ± 0,002590	0,09452 ± 0,002765	0,08987 ± 0,002763	0,08043 ± 0,003504
Goodness of Fit									
R square	0,980	0,990	0,988	0,991	0,988	0,991	0,989	0,988	0,976
Sy.x	0,053	0,038	0,042	0,037	0,040	0,033	0,035	0,035	0,045

Is slope significantly non-zero?									
F	646,3	1227	1031	1349	1105	1474	1169	1058	526,9
DFn, DFd	1,000, 13,00	1,000, 13,00	1,000, 13,00	1,000, 13,00	1,000, 13,00	1,000, 13,00	1,000, 13,00	1,000, 13,00	1,000, 13,00
P value	< 0,0001	< 0,0001	< 0,0001	< 0,0001	< 0,0001	< 0,0001	< 0,0001	< 0,0001	< 0,0001
Deviation from zero?	Significant	Significant	Significant	Significant	Significant	Significant	Significant	Significant	Significant
Runs test									
Points above line	2	3	2	2	2	3	3	3	2
Points below line	3	2	3	3	3	2	2	2	3
Number of runs	4	4	4	4	4	4	3	3	3
P value (runs test)	0,90	0,90	0,90	0,90	0,90	0,90	0,50	0,50	0,50
Deviation from linearity	Not Significant	Not Significant	Not Significant	Not Significant	Not Significant	Not Significant	Not Significant	Not Significant	Not Significant
		**BI9564 (µM) + 25 µM BAZ2-ICR**							
Best-fit values									
Slope	0,1048 ± 0,004124	0,1108 ± 0,004227	0,1133 ± 0,004851	0,1091 ± 0,004645	0,1098 ± 0,004772	0,1087 ± 0,003777	0,1026 ± 0,003611	0,09608 ± 0,003563	0,09371 ± 0,003394
Goodness of Fit									
R square	0,980	0,981	0,977	0,977	0,976	0,985	0,984	0,982	0,983
Sy.x	0,053	0,054	0,062	0,060	0,061	0,048	0,046	0,046	0,044
Is slope significantly non-zero?									
F	646,3	687,4	545,3	551,5	529,7	828,5	808,1	727,2	762,3
DFn, DFd	1,000, 13,00	1,000, 13,00	1,000, 13,00	1,000, 13,00	1,000, 13,00	1,000, 13,00	1,000, 13,00	1,000, 13,00	1,000, 13,00
P value	< 0,0001	< 0,0001	< 0,0001	< 0,0001	< 0,0001	< 0,0001	< 0,0001	< 0,0001	< 0,0001
Deviation from zero?	Significant	Significant	Significant	Significant	Significant	Significant	Significant	Significant	Significant
Runs test									
Points above line	2	2	2	2	2	2	2	3	3
Points below line	3	3	3	3	3	3	3	2	2
Number of runs	4	4	4	4	4	4	4	3	3
P value (runs test)	0,90	0,90	0,90	0,90	0,90	0,90	0,90	0,50	0,50
Deviation from linearity	Not Significant	Not Significant	Not Significant	Not Significant	Not Significant	Not Significant	Not Significant	Not Significant	Not Significant

Table S4. Analysis of fitness of the linear regression model for the growth curve of PC3 and 22Rv1 transfected with siRNA BAZ2A, RLUC and KIF11.

	**NT**	**siRLUC (neg ctrl)**	**siKIF11 (pos ctrl)**	**siBAZ2A**
**PC3**				
Best-fit values				
Slope	0,1656 ± 0,01088	0,1582 ± 0,008422	0,1031 ± 0,02350	0,1220 ± 0,01327
Goodness of Fit				
R square	0,959	0,973	0,658	0,894
Sy.x	0,086	0,067	0,187	0,105
Is slope significantly non-zero?				
F	231,9	353	19,24	84,57
DFn, DFd	1,000, 10,00	1,000, 10,00	1,000, 10,00	1,000, 10,00
P value	< 0,0001	< 0,0001	0,0014	< 0,0001
Deviation from zero?	Significant	Significant	Significant	Significant
Runs test				
Points above line	1	2	2	3
Points below line	3	2	2	1
Number of runs	3	4	4	3
P value (runs test)	1	1	1	1
Deviation from linearity	Not Significant	Not Significant	Not Significant	Not Significant
**22Rv1**				
Best-fit values				
Slope	0,1180 ± 0,006537	0,1091 ± 0,006126	0,09007 ± 0,004655	0,09135 ± 0,005985
Goodness of Fit				
R square	0,962	0,961	0,966	0,947
Sy.x	0,084	0,079	0,060	0,077
Is slope significantly non-zero?				
F	325,8	317,5	374,4	233
DFn, DFd	1,000, 13,00	1,000, 13,00	1,000, 13,00	1,000, 13,00
P value	< 0,0001	< 0,0001	< 0,0001	< 0,0001
Deviation from zero?	Significant	Significant	Significant	Significant
Runs test				
Points above line	3	3	3	2
Points below line	2	2	2	3
Number of runs	4	4	3	5
P value (runs test)	0,9	0,9	0,5	1
Deviation from linearity	Not Significant	Not Significant	Not Significant	Not Significant

Table S5. Analysis of fitness of the segmental linear regression model for the growth of spheroids formed by PC3 and 22Rv1 treated with fixed concentration of BAZ-ICR and GSK2801 (25 µM) and BI9564 (20 µM) alone and in combination with 25 µM BAZ2-ICR.

	**untreated**	**25 µM GSK2801**	**25 µM BAZ2-ICR**	**20 µM BI9564**	**20 µM BI9564 + 25 µM BAZ2-ICR**
**PC3**					
Segmental linear regression					
Best-fit values					
slope1	55146	5023	62282	28821	32279
X0	6	6	6	6	6
slope2	80371	19191	77288	69835	66679
Std. Error					
slope1	14191	5117	11511	6624	5258
slope2	7758	2937	6419	4299	3412
Goodness of Fit					
R square	0,8793	0,6142	0,8922	0,9467	0,9645
Sy.x	149542	62502	138290	70391	55868
Replicates test for lack of fit					
SD replicates	150642	68941	144876	83442	65113
SD lack of fit	146194	36981	115587	40862	35931
Discrepancy (F)	0,9418	0,2877	0,6365	0,2398	0,3045
P value	0,5017	0,9839	0,7849	0,9902	0,9756
Evidence of inadequate model?	No	No	No	No	No
Constraints					
X0	X0 = 6,000	X0 = 6,000	X0 = 6,000	X0 = 6,000	X0 = 6,000
**22Rv1**					
Best-fit values					
slope1	17538	13070	17642	12255	10407
X0	9	9	9	9	9
slope2	17870	13213	10618	6998	6613
Std. Error					
slope1	1046	664,2	778,9	1176	1147
slope2	426,8	308,3	381,2	439,3	423
Goodness of Fit					
R square	0,9678	0,9799	0,9633	0,9103	0,9005
Sy.x	29816	15894	20326	25231	24611
Replicates test for lack of fit					
SD replicates	31125	17827	21881	27696	27494
SD lack of fit	23166	9538	14616	16806	14317
Discrepancy (F)	0,554	0,2862	0,4462	0,3682	0,2712
P value	0,9513	0,9995	0,9872	0,9931	0,9992
Evidence of inadequate model?	No	No	No	No	No
Constraints					
X0	X0 = 9,000	X0 = 9,000	X0 = 9,000	X0 = 9,000	X0 = 9,000

Table S6. List of primers used in this study

**Gene name**		**sequence**
BAZA	FW	ATGGAAATGGAGGCAAACGAC
	RV	GAGACCCGTTAGTGTAGAGGC
BAZB	FW	AAGGTTTCCCAACAGTGATGTC
	RV	AAGCCACAGAAGGTGTCGAA
AR	FW	GGTGAGCAGAGTGCCCTATC
	RV	ATGGGCAAAACATGGTCCCT
EZH2	FW	TACTTGTGGAGCCGCTGAC
	RV	CTGCCACGTCAGATGGTG
ZFN185	FW	TCAGCGGCCAAGAAGAGC
	RV	ACTGTTTGAGCCAGGAGTGG
AOX1	FW	AAACGCCTCGAACCCATCAT
	RV	CCTTCGCCTTTCTCCCAGTT
HOMER2	FW	TTGACGCAGAGCGCAGCCAA
	RV	TGCAGTGCTGTGGTCAGCCG
HOXA7	FW	TGAGGCCAATTTCCGCATCT
	RV	TCGGACCTTCGTCCTTATGC
MKX	FW	TCGGCCTGAGACACCGGAGG
	RV	CGTCATCTGCGAGCCGAGGG
KFL6	FW	TGGAGGAGTACTGGCAACAG
	RV	TGAAACATAGCAGGGCTCGC
Housekeeping genes		
HPRT1	FW	CTCATGGACTGATTATGGACAGGAC
	RV	GCAGGTCAGCAAAGAACTTATAGCC
GAPDH	FW	TGCACCACCAACTGCTTAGC
	RV	GGCATGGACTGTGGTCATGAG
ACTB	FW	TGTACGCCAACACAGTGCTG
	RV	GCTGGAAGGTGGACAGCGA

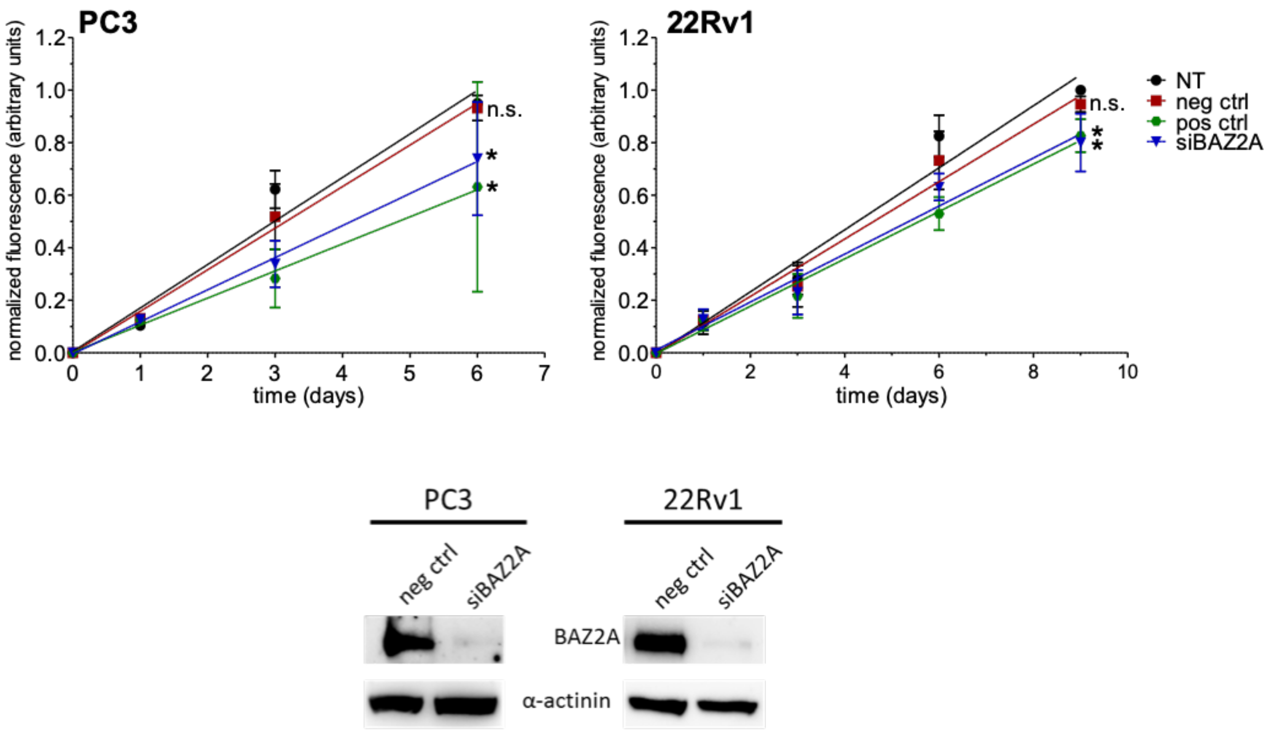

Figure S1. Effect of silencing BAZA through siRNA in PCa cells. The reduction of BAZ2A caused a decrease in the growth rate of both PC3 (left) and 22Rv1 (right), similar to the one observed when KIF11 (positive control) was silenced. Each point represents the mean ± SD of 3 independent replicates. For each replicate, three wells/condition were seeded. Lines are obtained using the linear regression analysis of the GraphPad Prism software. The treatment was considered to have an effect when the slopes of the treated conditions and the negative control were statistically different. It was first assessed that the non-transfected condition and the negative control (transfected with siRLUC) had no significant difference. ns = $P > 0.05$, $^*P < 0.05$. The figure includes the western blot analysis of BAZ2A in PCa cells. The total protein content was extracted from cells transfected with siRNA BAZ2A or siRNA RLUC (negative control). A loading control (α-actinin) was used to show that the same amount of protein lysate was loaded for the two conditions. The figure shows one out of three independent replicates. BAZ2A amount in PC3 is significantly lower than in 22Rv1 (exposure 430 sec vs. 29 sec in PC3 and 22Rv1, respectively). Transfection efficiency was also more variable in PC3 cells compared to its high consistency in 22Rv1.

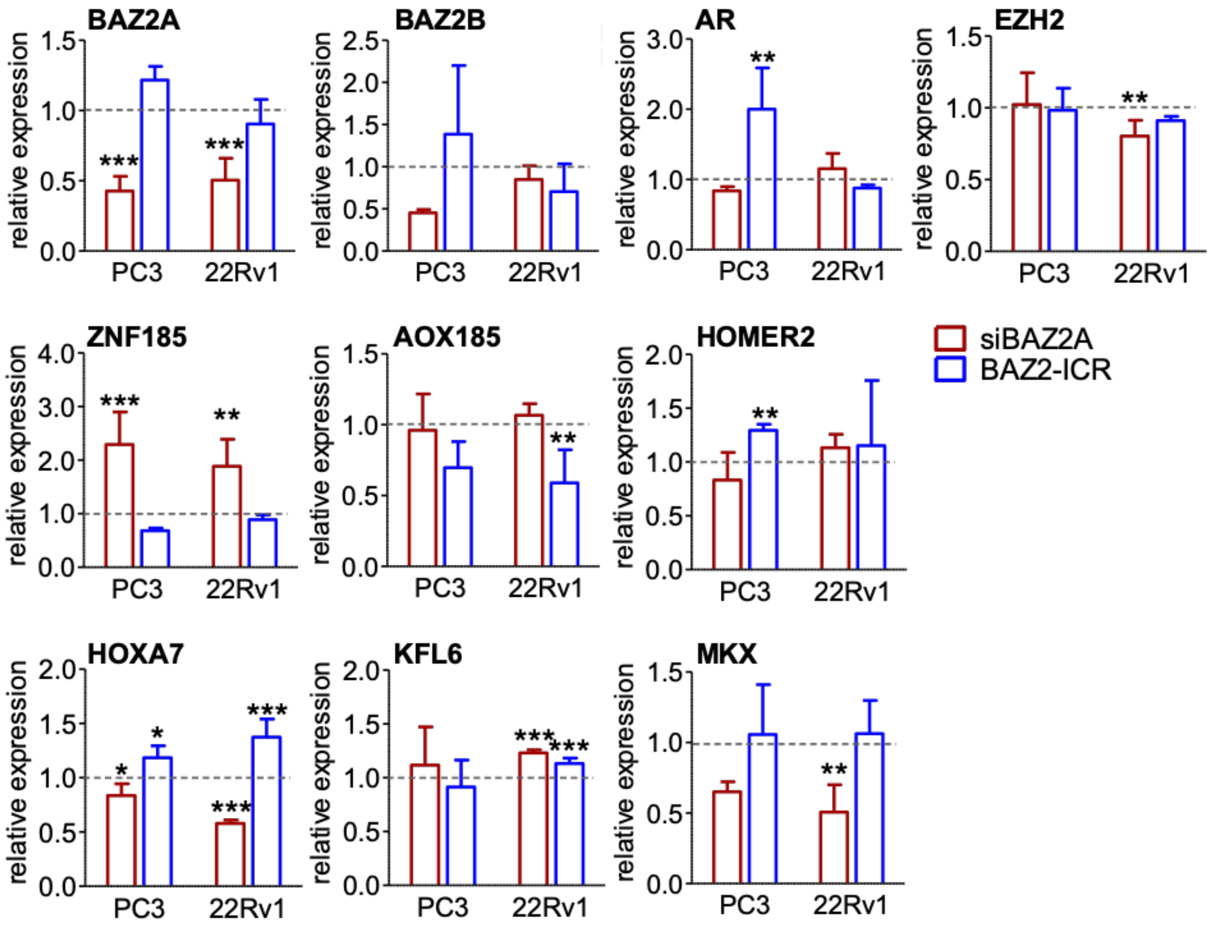

Figure S2. The graphs show the relative gene expression in PCa cells of BAZ2A, BAZ2B, AR, EZH2, ZNF185, AOX185, HOMER2, HOXA7, KFL6 and MKX. The RNA was extracted from untreated cells or cells treated for 6 days with 25 µM BAZ2-ICR and from cells transfected with siRNA BAZ2A or siRNA RLUC (negative control). The normalization for cells treated with BAZ-ICR was done using the values obtained from untreated cells, while the one for cells transfected with siRNA BAZA used the values obtained from cells transfected with siRNA RLUC. The control (untreated or negative control) is represented as a grey dotted line with the value 1.0 of relative expression. The graphs show the mean ± SD of 3 independent experiments. The statistical significance was evaluated with 1way Anova comparing the treatments to the respective controls. When not otherwise written, the difference resulted non-significant (ns = $P > 0.05$), *$P < 0.05$, **$P < 0.01$, ***$P < 0.001$.

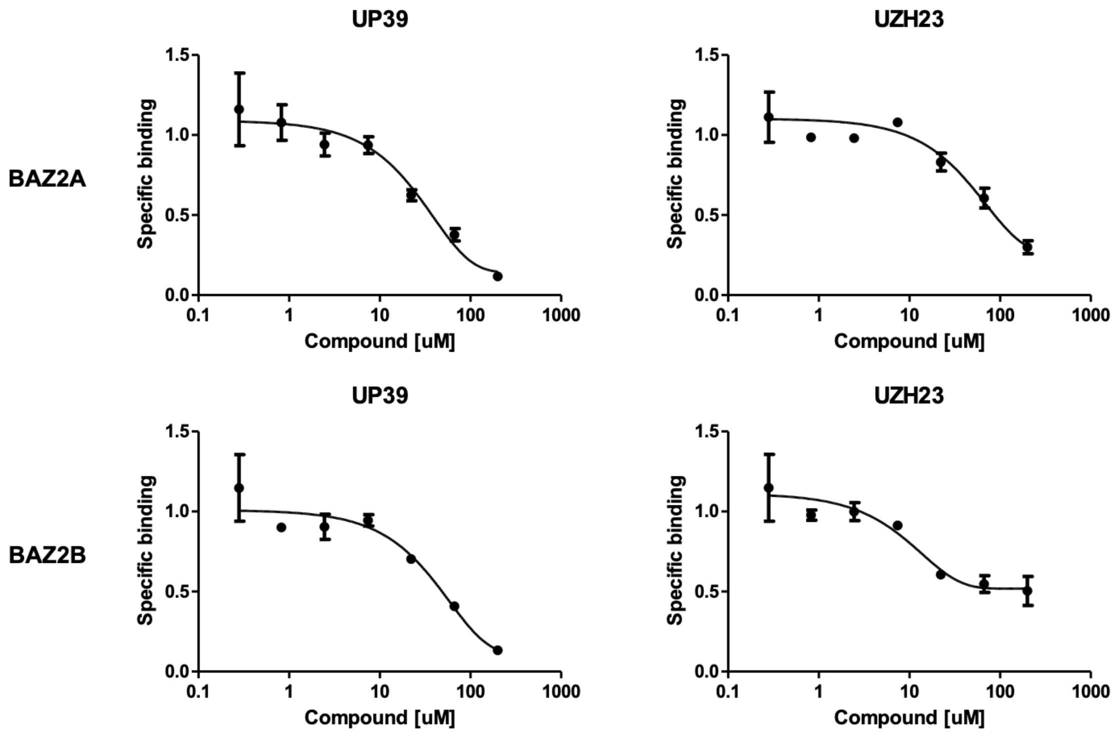

Figure S3. AlphaScreen competition binding assay for compounds UP39 and UZH23. The specific binding to the acetylated peptide relative to the control DMSO (y-axis) is plotted against the corresponding compound concentration in μM in log10 scale (x-axis).

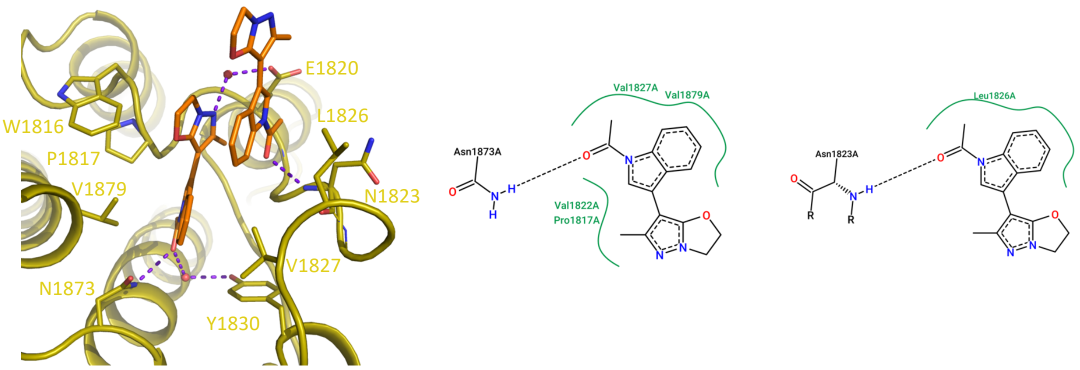

Figure S4. UZH23 in complex with BAZ2A: in this view, the four-layer sandwich involving W1816, the two UZH23 molecules, and Leu1826 can be appreciated together with the water-bridged interaction between the pyrazole-oxazole tail and Glu1820.

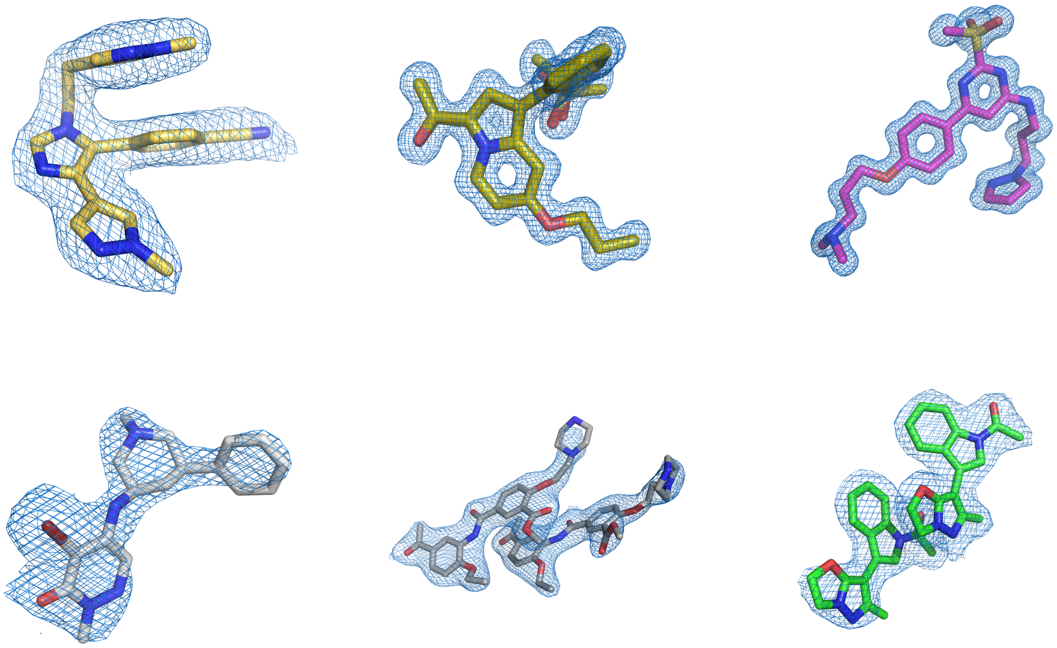

Figure S5. Electron densities for the analyzed compounds. $2F_o$-F_c map is contoured at 1σ for BAZ2-ICR (yellow), GSK2801 (gold), TP-238 (magenta), GSK4027 (white), UP39 (gray) and UZH23 (green).

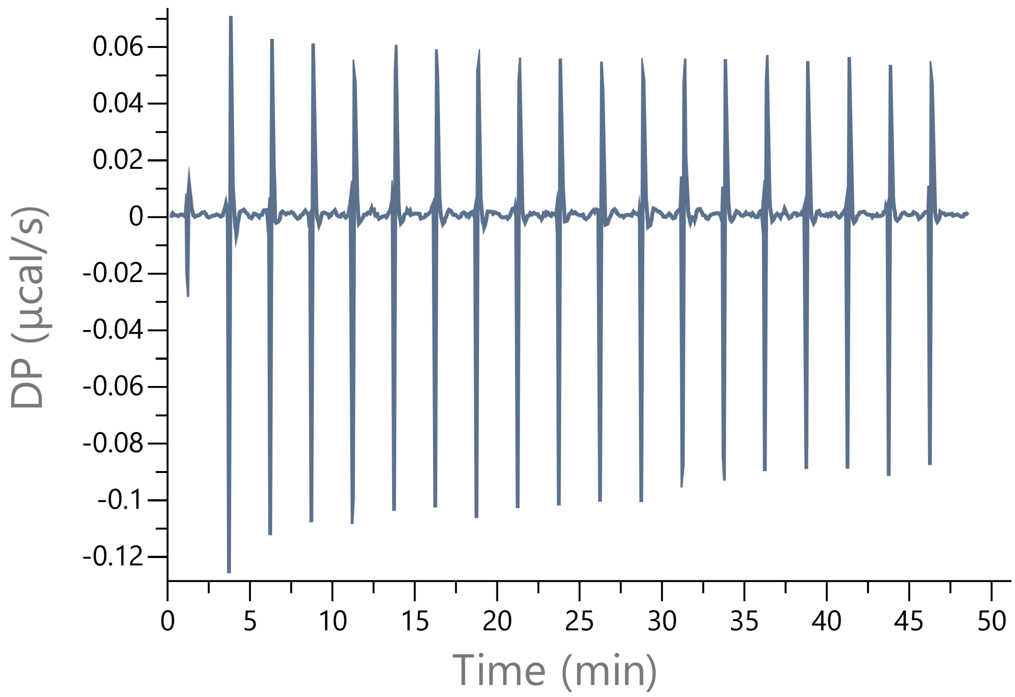

Figure S6. ITC blank titration.
